# The implementation of the urinalysis method for ^210^Po monitoring of employees in the selected industrial sector operating with NORMs in poland

**DOI:** 10.13075/ijomeh.1896.02688

**Published:** 2026

**Authors:** Katarzyna Rzemek, Małgorzata Dymecka

**Affiliations:** Radiation Protection Measurements Laboratory, National Centre for Nuclear Research, Otwock, Poland

**Keywords:** urine analysis, method validation, radiation exposure, alpha spectrometry, polonium, NORM

## Abstract

**Objectives:**

This study is focused on internal exposure to ^210^Po, a highly radiotoxic α-emitting radioactive element. This isotope is important in evaluating internal exposure among industrial workers, especially in sectors that handle naturally occurring radioactive materials (NORMs), where polonium can be present in workplace as a result of its release in thermal processes and other processes in which enhanced NORMs are utilized. The aim of this paper is to present a validation of an implemented method for determining ^210^Po in urine and its application in industrial sector where internal ^210^Po contamination is a potential risk.

**Material and Methods:**

The presented method for determination of ^210^Po activity in urine involved radiochemical preparation of 24-hour urine samples followed by spontaneous deposition of polonium on a silver disc and measurement by α spectrometry. The method was applied to urine samples analyzed as part of the validation process as well as to urine samples collected by industrial workers.

**Results:**

The method was validated in interlaboratory comparison and implemented in Radiation Protection Measurements Laboratory (Laboratorium Pomiarów Dozymetrycznych – LPD) of the National Centre for Nuclear Research in Poland. Precision, selectivity and detection limit were also characterized in validation process. The implemented method allowed to obtain relatively high values of tracer recovery (^209^Po), on average at a level >70%.

**Conclusions:**

Conducted analyses of urine samples collected from selected industrial workers confirmed that radiation exposure to ^210^Po at positions directly related to the technological process was higher than at other positions, however committed effective doses were on the safe level.

## Highlights

- Radiochemical assays are crucial for assessing internal α-emitter exposure.
- Urinalysis is an effective method for monitoring potential ^210^Po intakes.
- Polish industry workers handling naturally occurring radioactive materials are at potential risk of internal ^210^Po exposure.
- Quantifying the activity of ^210^Po in chemical reagents is of great importance.

## INTRODUCTION

Numerous materials applied across different sectors in the industry contain natural radioactive isotopes, mainly isotopes from the uranium and thorium series. These materials have become known as naturally occurring radioactive materials (NORMs). Most radiation from NORMs under normal circumstances does not pose significant health risk, however, certain human activities and technological processes in which NORMs are involved, may enhance the radiation exposure of workers and members of the public. During industrial processes, the activity concentration levels of naturally occurring radionuclides may increase significantly compared to the activity concentrations in the original material and their physicochemical properties may alter [1]. Such materials of enhanced radioactivity are often referred to as technologically enhanced naturally occurring materials (TENORMs). Mining and processing of ores, oil and gas recovery processes, the phosphate mining and processing, metal mining and processing (tin, copper, iron, steel, silver, gold, etc.) are the typical examples of work activities which may cause enhanced exposure [1–6]. The assessment of industrial workers’ internal exposure has become an important issue which has been discussed recently in scientific publications [3,7–9] and has also found reflection in the guidelines and regulatory frameworks.

A key legislation on the safety of persons who are occupationally exposed to ionizing radiation is the document established by the Council of the European Union, Directive 2013/59/EURATOM [10]. The Directive applies to, among other exposure situation, the area of processing materials containing naturally occurring radioactive elements which may increase workers potential exposure. This document establishes basic principles to protect health from radiological hazards. Poland has introduced its own legal regulations, which can be found in the amended Atomic Law Act which came into force in 2021 [11].

When considering internal contamination arising in industrial plants operating with NORMs and producing enhanced materials, the release of radioactive isotopes into the air during the thermal processes should be taken into account. This is particularly important in the case of isotopes with relatively low boiling points, such as polonium. In many metallurgical processes during metal extraction from the ore, which are carried out at high temperatures, like smelting and refining, ^210^Po may volatilize [1,6]. This isotope can be also precipitated and concentrated, according to the data presented by ICRP, up to 200 000 Bq/kg [1]. Processing of enhanced NORMs can lead to increased levels of dust in the workplace. This type of dust may pose a radiological hazard primarily due to 2 key features. Firstly, the dust particles can act as carriers for radioelements’ vapor condensate. Secondly, the dust itself may be composed of fine particles derived from the NORMs being processed. Mechanical treatment of materials, other dry processes, maintenance of scrubbers and filters are examples of work posing inhalation risk for the personnel [6,12].

Due to the fact that notable levels of ^210^Po activity, an isotope within the uranium series, can be observed in work environment of industries dealing with NORMs, it is crucial to take into account internal exposure to polonium in the occupational risk assessment. This highly radiotoxic isotope with a half-life of 138.4 days [13] emits α radiation and causes a significant radiation hazard when it enters the human body through inhalation, ingestion or body tissue. Alpha particles are characterized by a high linear energy transfer (LET) value, meaning that energy transfer to cells occurs over a short distance causing ionization with high linear density. Alpha-emitting isotopes may cause adverse effects to humans due to their high radiotoxicity and chemical toxicity. Therefore, methods for detecting internal contamination from these isotopes are acknowledged as significant [14,15]. The occupational health risk posed by polonium can be illustrated by the comparison of the dose coefficient e(g) of ^210^Po (defined as effective dose caused by the intake of 1 Bq into the body) with dose coefficient e(g) of one of the most radiotoxic β radioactive elements, e.g., ^90^Sr. The dose coefficients, e(g), for the inhalation of ^210^Po and ^90^Sr are documented as 1.1 μSv/Bq and 0.018 μSv/Bq [16,17], respectively, assuming an aerosol of 5 μm activity median aerodynamic diameter (AMAD) and material of moderate absorption (Type M). The e(g) coefficient for ^210^Po is 2 orders of magnitude greater than for ^90^Sr, which directly correlates to the magnitude of the estimated dose.

There are findings in the literature indicating raised levels of ^210^Po activity concentration in air in metallurgic industry in Poland. Elevated ^210^Po concentrations in air were observed during loading the furnace in the molding area (≤1.3 Bq/m^3^) and during the casting procedure (≤4.7 Bq/m^3^) as a result of release of dust [18]. Another study has been conducted on ^210^Pb activity during the process of recycling of selected waste in the metallurgical industry producing distilled lead, suggesting a possible risk to workers from exposure to ^210^Pb and ^210^Po [19]. Due to the observed increase in ^210^Po and/or ^210^Pb activity levels in the metallurgical industry in Poland, the need to implement suitable radiological monitoring method arose making the development of appropriate analytical methods important.

As part of the internal monitoring of ^210^Po exposure, analyses of urine samples collected over a 24-hour period are commonly performed. The analysis usually starts by wet acid digestion, may involve coprecipitation of the isotope of interest and ends with the preparation of the α source, which is usually done by performing spontaneous deposition of polonium on a silver disc. Multiple studies addressing the topic of polonium activity determination in urine could be found [20–28]. Some of the publications concern only ^210^Po analyses in the general population, because knowledge of ^210^Po activity in urine of none-exposed people enables the evaluation of background levels of this isotope and also allows the assessment of the contribution of ^210^Po to the dose received by humans by consumption.

This study aims to implement in Radiation Protection Measurements Laboratory (Laboratorium Pomiarów Dozymetrycznych – LPD) of the National Centre for Nuclear Research (Narodowe Centrum Badań Jądrowych – NCBJ), Otwock, Poland, and validate the method for determination of the ^210^Po activity in urine samples for the purpose of the internal exposure assessment of industrial workers. According to Polish regulations, measurements used to assess doses from radiation exposure should be performed by accredited laboratories, which have been granted accreditation by the national body appointed by Polish government. For this reason, the investigated procedure was validated for the purpose of submission for accreditation by Polish Centre for Accreditation (Polskie Centrum Akredytacji – PCA), Warsaw, Poland. The validation results are presented in this work.

The paper also discusses the ^210^Po activity content in reagents used in the analytical procedure increasing the background and proposes a calculation method that takes this influence into account. The described method was applied to conduct internal contamination monitoring among individuals employed in the industrial sector, selected results are also presented in this study.

## MATERIAL AND METHODS

### Reagents and standards

The tracer solution of ^209^Po (SRM 4326a) was purchased from National Institute of Standards and Technology, Gaithersburg, USA. The solution was diluted by the Radioisotope Center POLATOM at the NCBJ in Poland. The activity of 1 g of the prepared solution was 0.4891±0.0050 Bq (k = 2). The measurement silver discs with diameter of 25.1 mm and a thickness of 0.27 mm were bought from Mennica – Metale (Radzymin, Poland). All reagents used in the radiochemical procedures were of analytical grade.

### Tested samples

During the validation procedure, 3 types of samples were analyzed:

a) urine samples with the same^210^Po activity concentration
b) blank samples
c) urine samples from interlaboratory comparisons.

Analysis of a series of urine samples with the same ^210^Po con-centration (type “a” urine samples) allowed to determine the precision of the method. The analyses of blank samples (type “b” urine samples) were aimed at determining whether ^210^Po contamination from reagents was introduced during the analysis. Based on the performed measurements (including the α spectrometer background measurements), it was also possible to examine the selectivity of the method and the detection limit. In order to validate the method, analyses of samples from interlaboratory comparisons were carried out (type “c” urine samples).

The analysis of urine samples from people working in conditions of exposure to ^210^Po enabled the evaluation of the method's chemical efficiency. The study also presents a part of the results acquired through the individual monitoring of 3 selected industrial workers. Employees were instructed on the correct procedure for collecting 24-hour urine. To ensure the privacy and data confidentiality of employees data and biological samples, each employee had their own code, thus eliminating the need for identification using personal data (names). Employees filled out a form that included: employee code, date of sample collection and information regarding their place of employment. Samples were collected exclusively by men. Daily samples were collected in a plastic container and preserved by addition of nitric acid (65% HNO_3_). The samples were kept in a refrigerator until the radiochemical analyses were started.

### Radiochemical procedure

The procedure for the determination of ^210^Po was established in accordance with the methodology presented by Manickam et al. with some modifications [20]. The typical protocol requires the examination of 500 ml of a 24-hour urine sample. However, if smaller daily samples are available, which is probable for individuals employed in challenging industrial environments, whole daily urine sample can be taken to analysis.

Before sample preparation began, a known amount of polonium tracer (^209^Po) was added to each sample. The process of isolating polonium from a urine sample started with a 5-hour wet mineralization technique on a hot plate, which involved the use of 100 ml of 65% HNO_3_ and 100 ml of 35–38% HCl. Upon the cooling of the sample, through the addition of 500 ml of ultra-pure water, and adjusting the pH to 9, manganese dioxide was precipitated by the use of 5 ml 0.3 M MnCl_2_ and 0.2 M KMnO_4_. The following day, subsequent to the centrifugation of the precipitate, the sample was prepared for autodeposition. For this purpose, 9 g of 20% (weight per volume – w/v) NH_2_OH HCl solution in 0.5M HCl and 2 ml of 35–38% HCl were added to the sample. After the precipitate was dissolved, the sample was moved from centrifugation vessel to a glass beaker. This was followed by the addition of 40 ml of ultra-pure water, 1 ml of 20% (w/v) C_6_H_8_O_6_ solution in water and 6 g of 25% (w/v) Na_3_C_6_H_5_O_7_ solution in 0.5 M HCl, after which the sample was heated. The prepared solution was transferred do the PTFE autodeposition cell. Polonium was deposited onto a silver disc placed at the bottom of the cell. The autodeposition process was conducted at 85°C for 4 h with mechanical stirring (500 rpm).

### Alpha spectrometry

The α sources were measured in Alpha Analyst Model 7200 spectrometer (Canberra, Meriden, USA) with passivated implanted planar silicon (PIPS) detectors of 450 mm^2^ and 1200 mm^2^ active areas. The energy calibration of detectors was carried out using the calibration source containing α-emitting isotopes: ^238^U, ^234^U, ^239^Pu and ^241^Am (Eckert & Ziegler Analytics, Atlanta, USA). The spectrometer's stability was monitored by measuring the α calibration source in each chamber quarterly. The long-term evaluation involved documenting the data obtained from these measurements characterizing the stability of the system (including the peak area, centroid and a full width at half maximum [FWHM] from collected spectra) and their subsequent analysis.

## RESULTS

### Validation

The following section presents test results of the key parameters (including selectivity, precision and detection limit) that characterize a validated method. Additionally, participation in interlaboratory comparison was a crucial stage of the validation process, which is also shown below.

#### Selectivity

**Figure 1.**
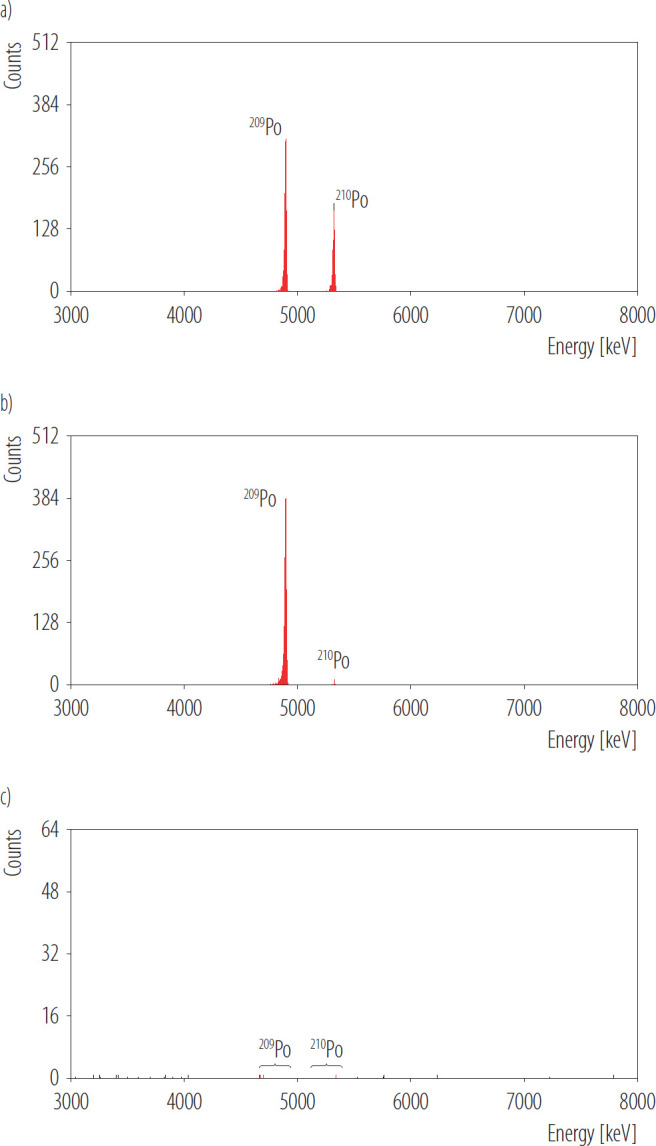
Alpha spectra of a) analyzed urine sample b) blank sample and c) silver disc obtained in Radiation Protection Measurements Laboratory of the National Centre for Nuclear Research in Poland, 2020

Analytical selectivity refers to the degree to which a method can accurately identify specific analytes within mixtures or matrices while avoiding interference from other components that exhibit similar characteristics [29]. The method for determining ^210^Po activity is considered selective if the isotope of interest and the tracer can be distinguished based on measurement. Additionally, any other α-emitting radioactive elements that might be present in the sample and could interfere with the polonium isotopes will be eliminated from the sample through chemical preparation. The selectivity of the polonium isotope determination method was assessed by analyzing the α spectra of 3 samples (Figure 1):

– a urine sample containing ^209^Po and ^210^Po isotopes,
– a blank sample (ultra-pure water with NaCl),
– a silver disc.

During the polonium determination method in urine, the spectrum from the prepared silver disc exhibited 2 characteristic peaks, confirming the presence of both the polonium tracer and ^210^Po (Figure 1a). The spectral peaks demonstrated FWHM <24 keV. The blank measurement revealed the presence of ^210^Po (Figure 1b). For this reason, the interference from ^210^Po in reagents in analytical samples must be taken into account. The presence of low activity of ^210^Po in the reagents does not affect the determination of ^210^Po activity in the samples if it is included in the calculations, however, it is associated with an increase in the detection limit. Analyses of blank samples without tracer (^209^Po) were also carried out. As a result of the measurements (measurement time of approx. 6 days), single counts (1–2 counts) were observed in the energy range for ^209^Po and a higher number of counts (60–70 counts) in the energy range for ^210^Po. In the spectrum obtained in the measurement of the silver disc (Figure 1c) peaks belonging to the ^209^Po and ^210^Po were not observed, single counts in a 7-day measurement were detected.

#### Precision

Precision refers to the closeness of the results obtained from repeated analyses using the same methodology. Precision can be characterized by “measurement repeatability” and “measurement reproducibility.” Repeatability refers to the degree of variation in results when a single analyst conducts measurements using the same equipment within a brief time frame. Reproducibility, which characterizes the greater variability of results, serves as an indicator of the differences in results across various laboratories [29]. In this study repeatability of the method was analyzed.

Precision was expressed by a statistical parameter – percentage coefficient of variation (CV) which was calculated based on standard deviation (SD). The precision of the method (repeatability) was assessed based on the results of measurement of 6 urine samples with the same ^210^Po activity concentration. Due to the fact that certified reference solution of ^210^Po was not available, the samples were obtained by combining several urine samples collected by persons working in conditions of potential exposure to internal ^210^Po contamination. The activity concentration of ^210^Po in the sample was approx. 0.022 Bq/l. The results of calculated ^210^Po activity concentrations in the tested samples are presented in Table 1. The percentage CV was calculated using the equation:

(1) $$\text{CV}=\frac{\text{SD}}{\overset{―}{\text{C}}}\times 100\%$$

where:

SD – standard deviation of obtained results (Bq/l),
$\bar{\text{C}}$ – mean of obtained results of ^210^Po activity concentration C_i_(Bq/l).

**Table 1.** Assessment of the precision of the method for ^210^Po determination in urine carried out in Radiation Protection Measurements Laboratory of the National Centre for Nuclear Research in Poland, 2020, during the validation procedure

Variable	Activity concentration C_i_ [Bq/l]	Activity concentration $\bar{\text{C}}$ [Bq/l] (M±SD)	Coefficient of variation [%]
Sample		0.0217±0.0017	7.6
1	0.0224		
2	0.0234		
3	0.0229		
4	0.0220		
5	0.0195		
6	0.0197		

As shown in Table 1, the percentage CV was 7.6%.

#### Minimum detectable activity concentration

Minimum detectable activity concentration (MDC) refers to the smallest concentration of an analyte that can be identified by a method (with a defined level of confidence) [29]. It was calculated according to the equation:

(2) $$\text{MDC}=\left(4.66\times \sqrt{\left({\text{N}\prime }_{210_{\text{Po}/\text{blank}}}+{\text{B}}_{210_{\text{Po}}}\times \text{t}\right)}+3\right)\times \frac{{\text{A}}_{\text{tr}}}{\text{V}\times \left({\text{N}}_{\text{tr}}-{\text{B}}_{\text{tr}}\times \text{t}\right)}$$

where:

N’_^210^Po/blank_ – net counts of the peak belonging to the ^210^Po in the blank sample, assuming that the blank measurement conditions are identical to those for the analytical sample,
B_^210^Po_ – background counting rate in the energy range of α particles emitted by ^210^Po determined for each detector (ratio of the number of pulses to the measurement time of the silver disc) (1/s),
t – measurement time (s),
A_tr_ – activity of the tracer (Bq),
N_tr_ – counts of the peak belonging to the tracer,
B_tr_ – background counting rate in the energy range of α particles emitted by ^209^Po determined for each detector (1/s),
V – volume of urine used for analysis from a 24-hour urine sample (l).

The value of N’_^210^Po/blank_ was determined by the formula:

(3) $${\text{N}}_{210_{\text{Po}/\text{blank}}}^{\prime }=\text{K}\times \frac{\left({\text{N}}_{\text{tr}}-{\text{B}}_{\text{tr}}\times \text{t}\right)}{{\text{A}}_{\text{tr}}}$$

where:

K – constant value determined for a series of blank samples (Bq).

Parameter K was calculated according to:

(4) $${\text{K}}_{\text{i}}=\frac{\left({\text{N}}_{210_{{\text{p}}_{\text{o}/{\text{blank}}_{\text{i}}}}}+{\text{B}}_{210_{{\text{p}}_{\text{o}}}}\times {\text{t}}_{{\text{blank}}_{\text{i}}}\right)\times {\text{A}}_{\text{tr}/{\text{blank}}_{\text{i}}}}{\left({\text{N}}_{\text{tr}/{\text{blank}}_{\text{i}}}-{\text{B}}_{\text{tr}}\times {\text{t}}_{{\text{blank}}_{\text{i}}}\right)}$$

where:

N_^210^Po/blank_i__ – counts of the peak belonging to the ^210^Po in the blank sample,
N_tr/blank_i__ – counts of the peak belonging to the tracer in the blank sample,
A_tr/blank_i__ – activity of the tracer added to the blank sample (Bq),
t_tr/blank_i__ – measurement time of blank sample [s].

The equation for determining the value of N’_^210^Po/blank_ takes into account the influence of ^210^Po activity content in reagents on the measurement of ^210^Po activity in analytical samples. This value was calculated for each measured sample based on the measurement of the blanks (ultra-pure water with the addition of NaCl) and was normalized to the measurement conditions for analytical samples (counting time, tracer recovery, counting efficiency). An alternative approach to account for the impact of ^210^Po from reagents when measuring ^210^Po in analytical samples involves measuring the activity concentration of ^210^Po in blank samples and then subtracting this value from the measured concentration of polonium in the analytical sample. It has been confirmed that both methods used to consider the effect of reagents-derived ^210^Po lead to analogous results. In this work, the method of determining the parameter K was applied. The average MDC value determined for samples measured in 2021–2024 (770 samples) taking into account the impact of ^210^Po from reagents was 0.00098 Bq/l. The MDC values for blank samples analyzed in 2021–2024 were in the range of 0.0002–0.0004 Bq/l for measurement times of 4–7 days (in the formula of MDC, the part N’_^210^Po/blank_ was eliminated).

#### Interlaboratory comparisons

The validation of the method was carried out in 2020 by participation in international comparisons organized by the French Association for the Promotion of Quality Control in Radiotoxicological Analysis (PROCORAD) in the field of determining ^210^Po activity in urine. In 2023, LPD took part in this comparison once again. In these comparisons, ^210^Po was determined in 0.5 l urine samples, labeled 20POA and 23POA, which were obtained in 2020 and 2023, respectively. It was defined that the activity concentration of ^210^Po was <1 Bq/l in the sample 20POA and <2 Bq/l in the sample 23POA. The organizers of intercomparison assessed the results provided by the laboratories using the parameters for evaluating quantitative data: z-score, bias (δ_x_) and E_n_ number. The selected performance indicator was z-score, whereas bias (δ_x_) and E_n_ were provided for information purposes.

Table 2 includes the equations used to calculate statistical parameters as well as the acceptance criteria for verifying results.

**Table 2.** Performance criteria in PROCORAD interlaboratory comparisons organized in 2020 and 2023

Parameter	Equation	Satisfactory result
Z-score	$z=\frac{C_{\text{lab}}-C_{\text{ref}}}{\hat{\sigma }}$	<2
Bias (δ_x_)	$\delta_{x}=\frac{C_{lab}-C_{ref}}{C_{ref}}\times 100\text{\%}$	∈(−25%, 50%)
E_n_ number	$E_{n}=\frac{C_{lab}-C_{ref}}{\sqrt{U_{lab}^{2}+U_{ref}^{2}}}$	<1

C_lab_ – laboratory's result (Bq/l); C_ref_ – assigned value (Bq/l); $\hat{\sigma }$ – robust standard deviation (Bq/l); U_lab_ – expanded uncertainty (k = 2) on the laboratory's result (Bq/l); U_ref_ – expanded uncertainty (k = 2) on assigned value (Bq/l).

The laboratory determined the ^210^Po activity in both samples, 20POA and 23POA, according to the presented method. The activity concentration of ^210^Po in the sample was calculated as follows:

(5) $${\text{C}}_{\text{lab}}=\frac{\left({\text{N}}_{210_{\text{po}}}-{\text{N}}_{210_{\text{po}/\text{blank}}}^{\text{'}}-{\text{B}}_{210_{\text{po}}}\times \text{t}\right)\times {\text{A}}_{\text{tr}}}{\left({\text{N}}_{\text{tr}}-{\text{B}}_{\text{tr}}\times \text{t}\right)\times \text{V}}$$

where:

C_lab_ – laboratory's result (Bq/l),
N_^210^Po_ – counts of the peak belonging to the ^210^Po.

The combined relative uncertainty (u_lab_) of the determined activity concentration of the isotope ^210^Po takes into account the following components:

– the relative uncertainty of counts in the peak originating from the ^210^Po isotope (including the influence of ^210^Po from the blank sample),
– the relative uncertainty of counts in the peak originating from the tracer,
– the relative uncertainty of the tracer's activity,
– the relative uncertainty of the mass of the tracer solution taken (considering the mass of the solution before and after sampling),
– the relative uncertainty of the determined activity concentration of ^210^Po in the blank sample.

The expanded uncertainty (U_lab_), expressed at approx. 95% confidence level (Cl) using a coverage factor of k = 2, was determined based on the relationship:

(6) $${\text{U}}_{\text{lab}}=2\times {\text{u}}_{\text{lab}}\times {\text{C}}_{\text{lab}}$$

The results obtained by LPD, the reference values and statistical parameters are presented in Table 3.

**Table 3.** Determination of ^210^Po activity in urine samples from intercomparisons organized by PROCORAD in 2020 (20POA) and 2023 (23POA) – results obtained by Radiation Protection Measurements Laboratory of the National Centre for Nuclear Research in Poland, and their performace

Sample	C_lab_±U_lab_ [Bq/l]	C_ref_± U_ref_ [Bq/l]	Z-score	Bias (δ_x_) [%]	E_n_
20POA	0.475±0.12	0.490±0.023	–0.1	–3.1	–0.1
23POA	0.722±0.11	0.651±0.036	0.6	11	0.6

C_lab_ – laboratory's result (Bq/l); C_ref_ – assigned value (Bq/l); U_lab_ – expanded uncertainty (k = 2) on the laboratory's result (Bq/l); U_ref_ – expanded uncertainty (k = 2) on assigned value (Bq/l).

### Urine samples

The following section refers to the analysis of 24-hour urine samples collected by workers potentially exposed to internal contamination from ^210^Po. The section provides the description of a parameter that characterizes the implemented method, the tracer recovery value. Part of the results obtained from the individual monitoring of 3 selected industrial workers are subsequently shown. The staff being monitored were employed within the same organizational unit, and their positions were associated with varying levels of exposure. The presented results were obtained for the individuals working at the positions associated with one of the highest (position A and B) and one of the lowest (position C) levels of exposure.

#### Tracer recovery

In analytical methods using tracers, it is not required to calculate the tracer recovery (chemical yield) to determine the activity concentration of the isotope being tested. Activity concentration of ^210^Po can be calculated due to ratio of the areas of the peaks of ^209^Po and ^210^Po [30]. However, assessing the tracer recovery is important for ensuring the quality control of the conducted analyses. In order to monitor the quality of the performed analyses, the tracer recovery R were determined according to the equation:

(7) $$\text{R}=\frac{{\text{N}}_{\text{tr}}}{\text{t}\times \text{E}\times {\text{A}}_{\text{tr}}}$$

where:

E – counting efficiency of detector.

**Figure 2.**
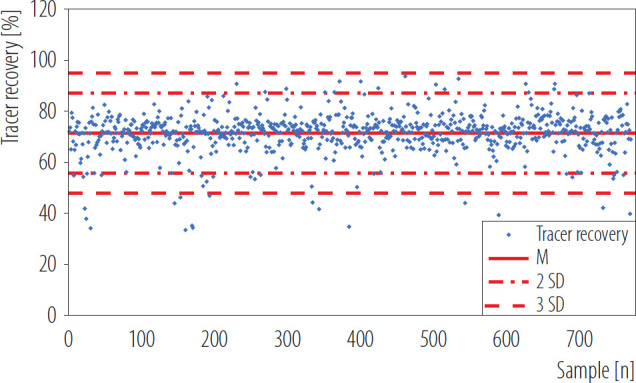
Recovery of ^209^Po tracer for analyses performed in Radiation Protection Measurements Laboratory of the National Centre for Nuclear Research in Poland, 2021–2024

Figure 2 shows the determined R values for the performed analyses of ^210^Po activity as part of the internal exposure monitoring of industrial workers carried out in 2021–2024, calculated on the basis of ^209^Po tracer measurements. The value of polonium tracer recovery was M±SD 71.4±7.9%.

#### Individual monitoring

Urine samples were collected from 2 workers of refinery, who were engaged in various tasks including loading the furnace in the molding section and casting procedure, where molten metal was transformed into solid object (position A and position B). During these processes elevated ^210^Po concentrations in air were observed due to the release of dust particles which behave as a carrier for ^210^Po and can pose a risk to workers [18]. The third employee was not directly involved in technological processes (position C). The volumes of 24-hour urine samples collected by employees at positions A, B, and C ranged 1.40–3.00 l (500 ml of the each daily sample was taken for radiochemical analysis). During the analyzed period (2021–2022), each employee provided 6 daily urine samples. The presented results of determined excreted ^210^Po activities in urine (A^i^, expressed in Bq/d) are normalized to median (Me) value of excreted ^210^Po activities (A_Me_, expressed in Bq/d) calculated for workers of the same department who collected 86 urine samples during this period. The contribution of ^210^Po activity from diet was not taken into account when calculating the ratio of A^i^/A_Me_.

**Figure 3.**
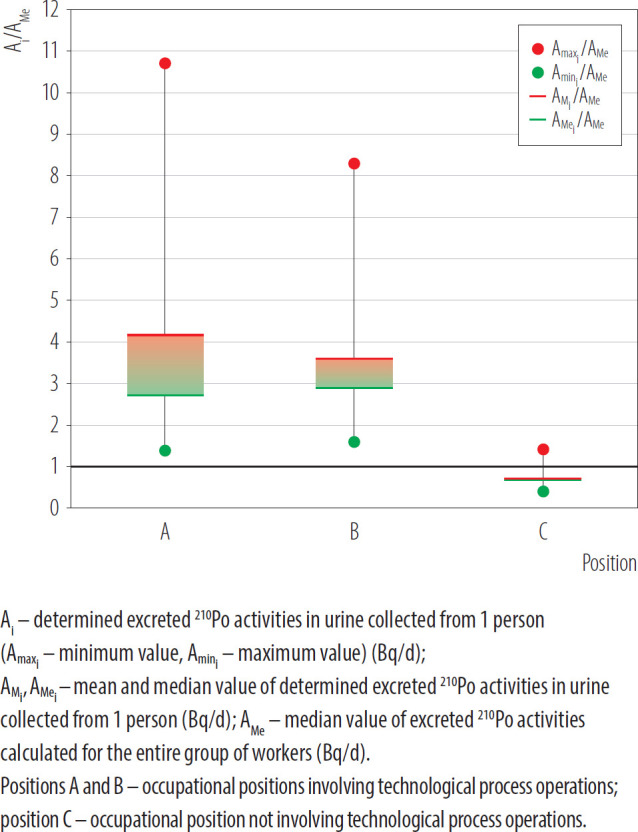
Results of excreted ^210^Po activity (A_i_/A_Me_) in urine for 3 metallurgical industry workers at position A, B and C obtained in Radiation Protection Measurements Laboratory of the National Centre for Nuclear Research in Poland, 2021–2022

The plot of Figure 3 illustrates the results derived from individual monitoring of 3 employees working at a refinery.

## DISCUSSION

### Validation

According to the presented results, the method demonstrates satisfactory selectivity. Analyzing the spectrum presented in Figure 1a, it can be concluded that as a result of the performed preparation, purified sources are obtained (there are no peaks from other isotopes in the spectrum). The method allows for selective separation of isotopes ^209^Po and ^210^Po from the matrix, the identification of which is possible in the measurement in an α spectrometer. The obtained peaks are characterized by good energy resolution (FWHM <24 keV). Analysis of the spectra obtained as a result of blank measurements revealed the presence of ^210^Po originating from the reagents used in the procedure rather than contamination from laboratory glassware or Teflon vessels, as no elevated counts were detected for the ^209^Po tracer used in each analysis. Based on the spectrum obtained in the measurement of the silver disc (Figure 1c), it can be observed that the background in the α spectrometer is very low, especially in the energy ranges corresponding to the energies of α particles of ^209^Po and ^210^Po. Considering the precision of the method, the percentage CV shown in Table 1 was found to be low, with a value of 7.6%. This value indicates that the method exhibits satisfactory precision. The obtained CV value is comparable to the value presented in the literature, which is approx. 10% for low ^210^Po activities in urine [20]. Described method for ^210^Po determination in urine is also characterized by a low MDC value, <0.001 Bq/l, which is in accordance with international standards [16]. The laboratory's results of the 2 urine samples analyzed in interlaboratory comparison (PROCORAD 2020, PROCORAD 2023) were satisfactory, the parameters determined by the organizers: the z-score, bias (δ_x_) and the E_n_ number for the results obtained by LPD were much lower than their critical values (as shown in Table 3). This proved the correct determination of the ^210^Po activity in the tested samples and a well-conducted uncertainty estimation procedure.

### Urine samples

Analyses of urine samples performed in 2021–2024 (Figure 2) indicate that the mean (M) value and standard deviation (SD) of polonium tracer recovery (R = 71.4±7.9%) is comparable to the values presented in the literature [20,21,23,28]. In the study of Manickam et al. [20] mean chemical efficiency combined for both rapid and sensitive method (methods differed, e.g., in the initial sample volume) of M±SD 85±10% was achieved. Obtaining a higher value could be related to pH value which was adjusted to 1.5–2.0 before polonium deposition stage. In another work by Meli et al. [21] the reported mean chemical yield was 60±14%. Lower value can be caused by the fact that the procedure without a coprecipitation step was applied.

Based on the analysis of urine samples collected from employees of industrial sector (Figure 3), it can be concluded that the highest ^210^Po activity in urine was found for the person at position A, which was approx. 11 times greater than A_Me_ calculated for urine samples collected from the entire group of workers. In the case of individuals employed at positions A and B, the mean values of ^210^Po activity in 24-hour urine samples were approx. 3–4 times the A_Me_ for the group. In urine samples collected by the individual at position C, the activities level of ^210^Po were at the level of A_Me_. Conducted analyses confirmed that radiation exposure to ^210^Po at position C is lower compared to work positions directly related to the technological process (positions A and B).

Effective dose assessments for occupationally exposed individuals were performed utilizing the data derived from polonium monitoring in urine samples. Internal dose assessments were conducted using the IMBA Professional Plus software that incorporated committed effective dose coefficients in accordance with Polish regulations [31]. The following scenario was adopted for dose assessment: chronic exposure, inhaled particulate materials (5 µm AMAD) Type M.

## CONCLUSIONS

The assessment of internal contamination from α-emitting isotopes is an important aspect in the case of individuals occupationally exposed to ionizing radiation. Radioanalytical method that enables the evaluation of human exposure to ionizing radiation, especially in case of α isotope intakes, is an important component for advancing this field. In this paper a radiochemical technique for determining ^210^Po in urine samples has been introduced on the basis of literature studies, which allows the assessment of internal exposure to this isotope.

The method for determining ^210^Po in urine samples has been validated in international interlaboratory comparisons and is characterized by, among others, satisfactory precision, high chemical efficiency and low minimum detectable activity concentration, which was proven in validation protocol. Based on the obtained results, it can be concluded that the presented method is suitable for the intended use.

The method was applied to perform analyses of urine samples collected from workers of the industry where NORMs are processed. Analyses of urine samples collected from industrial workers confirmed, that a correlation between radiation exposure to ^210^Po and the position of industrial workers within a technological process can be identified. Individuals engaged in industrial operations face higher levels of exposure. The effective doses in case of each of the studied groups of employees did not exceed the limit value. However, due to the occurrence of increased ^210^Po activity in the workplace, performing the individual monitoring of employees is advisable.

The described method for determining ^210^Po in urine samples can be applied in various areas of human activity, not limited to industries that utilize NORM materials, where a risk of internal contamination from polonium may exist. Currently studies are undertaken to apply the method of ^210^Po determination in urine to the radiological risk assessment from ^210^Pb.
